# Modified Two-Step Impressions via the Incorporation of Venting Holes: A Technical Report

**DOI:** 10.7759/cureus.64642

**Published:** 2024-07-16

**Authors:** Ahmed Yaseen Alqutaibi, Hatem Hazzaa Hamadallah, Mohamed M Rahhal, Wafaa Ibrahim Ibrahim

**Affiliations:** 1 Substitutive Dental Sciences (Prosthodontics), College of Dentistry, Taibah University, Al-Madinah Al-Munawwarah, SAU; 2 Prosthodontics, College of Dentistry, Ibb University, Ibb, YEM; 3 Orthodontics and Dentofacial Orthopedics, College of Dentistry and Hospital, Taibah University, Al-Madinah Al-Munawwarah, SAU; 4 Restorative Dentistry, Missouri School of Dentistry and Oral Health-A.T. Still University, Kirksville, USA; 5 Prosthodontics, Faculty of Oral and Dental Medicine, Fayoum University, Fayoum, EGY; 6 Prosthodontics, Faculty of Oral and Dental Medicine, Delta University for Science and Technology, Gamasa, EGY

**Keywords:** dimensional stability, dental technique report, margin accuracy, dental technique, venting holes, two-step impressions

## Abstract

This study presents a methodology for obtaining a precise impression of the crown margin of prepared teeth by utilizing a two-step impression technique. The method begins with the fabrication of a custom tray made from heat-cured acrylic resin, followed by the acquisition of an initial impression using putty elastomeric material. Subsequently, the impression is relieved around the prepared teeth, and vent holes are strategically placed through both the impression and the tray. Finally, a light-body consistency impression material is applied. This streamlined technique enhances efficiency and minimizes the errors commonly encountered with traditional two-step impression methods.

## Introduction

A precise impression is crucial for fabricating well-fitted and durable dental prosthetics. The capture of even the most minute details plays a vital role in producing superior dental restorations when making an impression [1]. In the case of crowns and bridges, two methods are commonly employed: the one-step method and the two-step method. The one-step method, which involves a heavy body material and a lighter consistency wash, is frequently utilized [2]. On the other hand, the two-step method begins with creating an initial impression using putty, a heavy or medium body material. Once removed from the mouth, a light body wash is applied, and the tray is repositioned inside the mouth [3]. The two-step method is generally considered more dimensionally accurate than the one-step method [4]. However, it does have several disadvantages. These include the potential for saliva to contaminate the putty, which can affect the adhesion of the light body to the impression, difficulties in repositioning the set putty in the mouth, increased chair time for the patient, and the requirement for a greater amount of materials [3].

Furthermore, studies have reported that the two-step method is more susceptible to errors when compared to the one-step technique [2,5]. The hydraulic and hydrophobic (H&H) technique was developed as a solution to the inaccuracies observed in the conventional two-step impression method [6]. This technique is recommended for accurately capturing the margins of crown preparations without the need for gingival retraction. The procedure begins by taking a putty impression without using a spacer or making any modifications to the impression. Subsequently, a light-body material is applied on top of the putty, and the patient is instructed to bite down again. As there is no space for the light body, hydraulic pressure is generated as the material is forced out from the preliminary impression, pushing it into the sulcus both laterally and occlusally [6]. However, a drawback of the H&H technique is that the reseating of the tray can cause the light body to exert stress on the set high-viscosity material, potentially distorting the impression [7]. Considering the limitations of the conventional two-step impression technique, this study aims to introduce a modified two-step impression methodology that seeks to simplify the process, minimize errors, and improve the accuracy of capturing crown margin preparations. By providing a systematic and concise guide, this technique strives to enhance the dimensional accuracy of impressions, reduce the risk of contamination and distortion, and, ultimately, ensure more reliable and well-fitting dental restorations.

## Technical report

**Figure 1:**
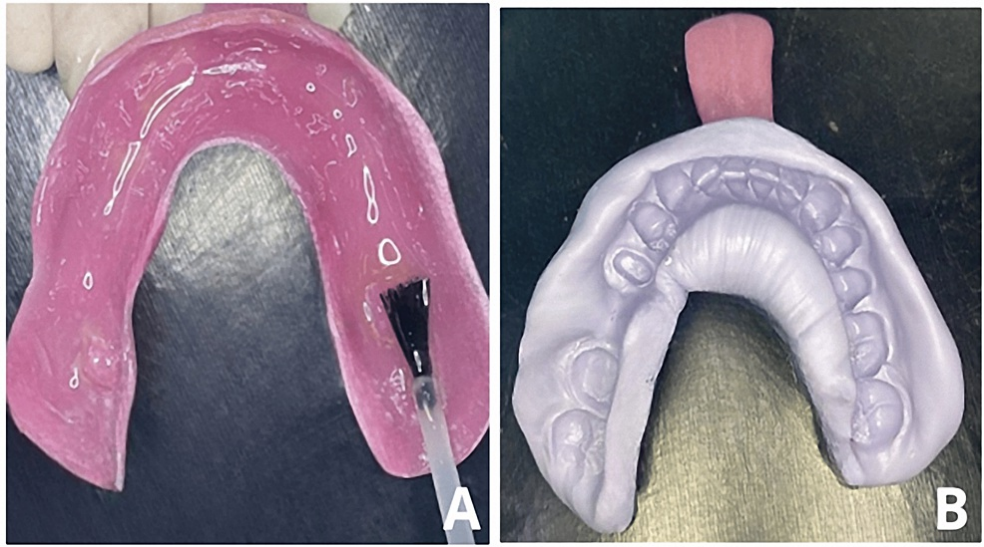
(A) Application of the adhesive to the fitting surface of the tray. (B) impression with putty consistency polyvinyl siloxane material.

The proposed modified two-step impression technique necessitates precise execution of the following steps to ensure optimal accuracy and detail in dental impressions. By following this method, practitioners can achieve superior results in capturing the intricate anatomical features necessary for effective diagnosis and treatment planning. The steps are as follows:

1. Make the primary impression using an irreversible hydrocolloid (Alginoplast, Kulzer®).

2. Utilize type III gypsum (Moldasynt, Kulzer®) to fabricate the dental cast.

3. Place two thicknesses of baseplate wax (modeling wax, Vertex Dental®) on the teeth.

4. Place a single layer of baseplate wax on the alveolar ridge to ensure sufficient room for the impression material.

5. Construct a custom, solid tray out of heat-cured acrylic resin( Regular, Vertex® Holland) with an attached handle.

6. Coat the fitting surface of the custom tray with a tray adhesive (VPS tray adhesive, 3M®).

7. Allow the adhesive to set for 7-15 minutes (Figure 1).

8. Insert a gingival retraction cord impregnated with a hemostatic agent (SureCord, Sure endo®, Gyeonggi-do, South Korea) into the gingival crevice around the teeth to be molded.

9. Carefully remove the gingival retraction cord.

10. Fill the tray with a putty-like polyvinyl siloxane (PVS) impression material (Variotime Easy Putty, Kulzer®).

11. Position the tray firmly over the area until the material sets completely (Figure 1).

12. Using a scalpel, carefully trim the set putty impression around each prepped tooth to eliminate undercuts and ensure even spreading of the final impression material (Figure 2).

**Figure 2:**
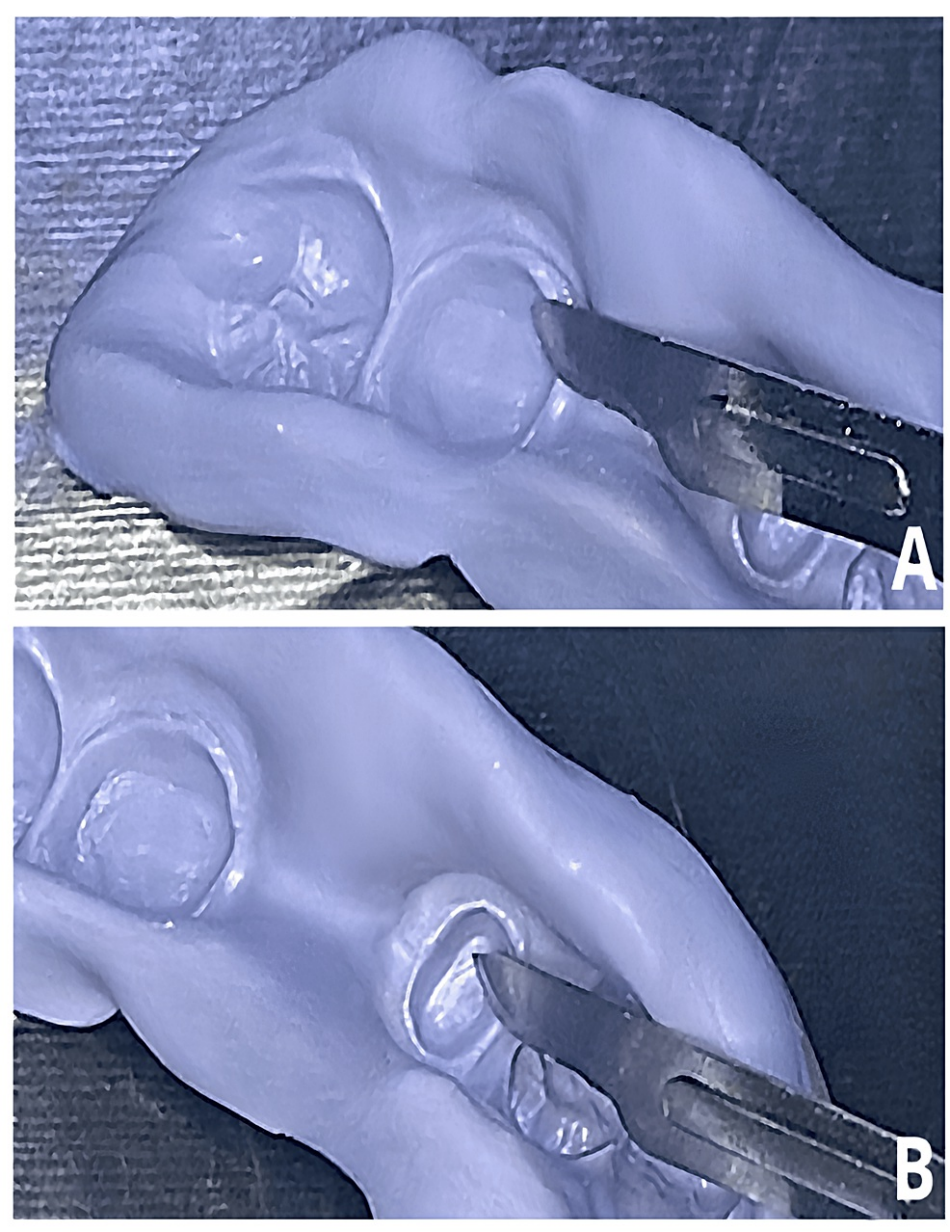
The putty impression is relieved with a scalpel around all prepared teeth to remove undercuts and allow even distribution of the final wash material.

**Figure 3:**
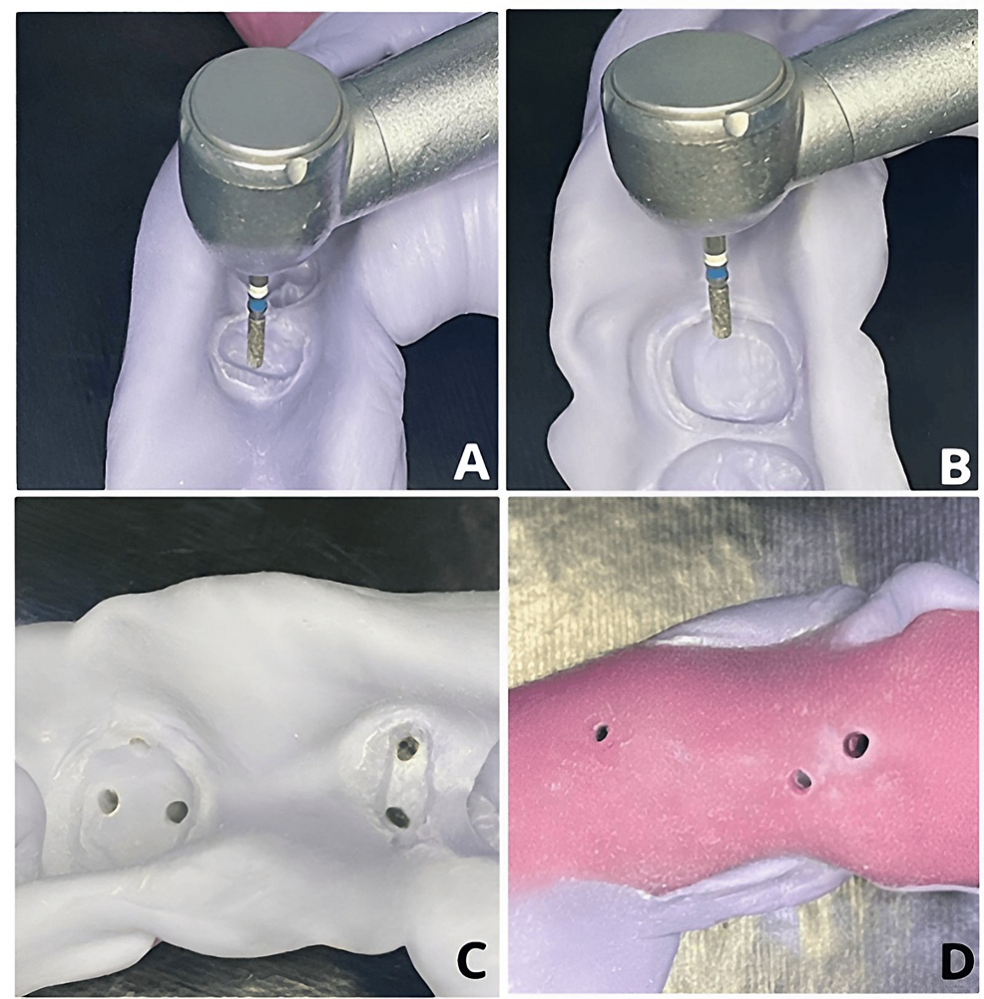
(A, B) Preparation of vent holes through the tray and the impression. (C) Observation of the venting holes through the impression. (D) Observation of the venting holes in the tray.

**Figure 4:**
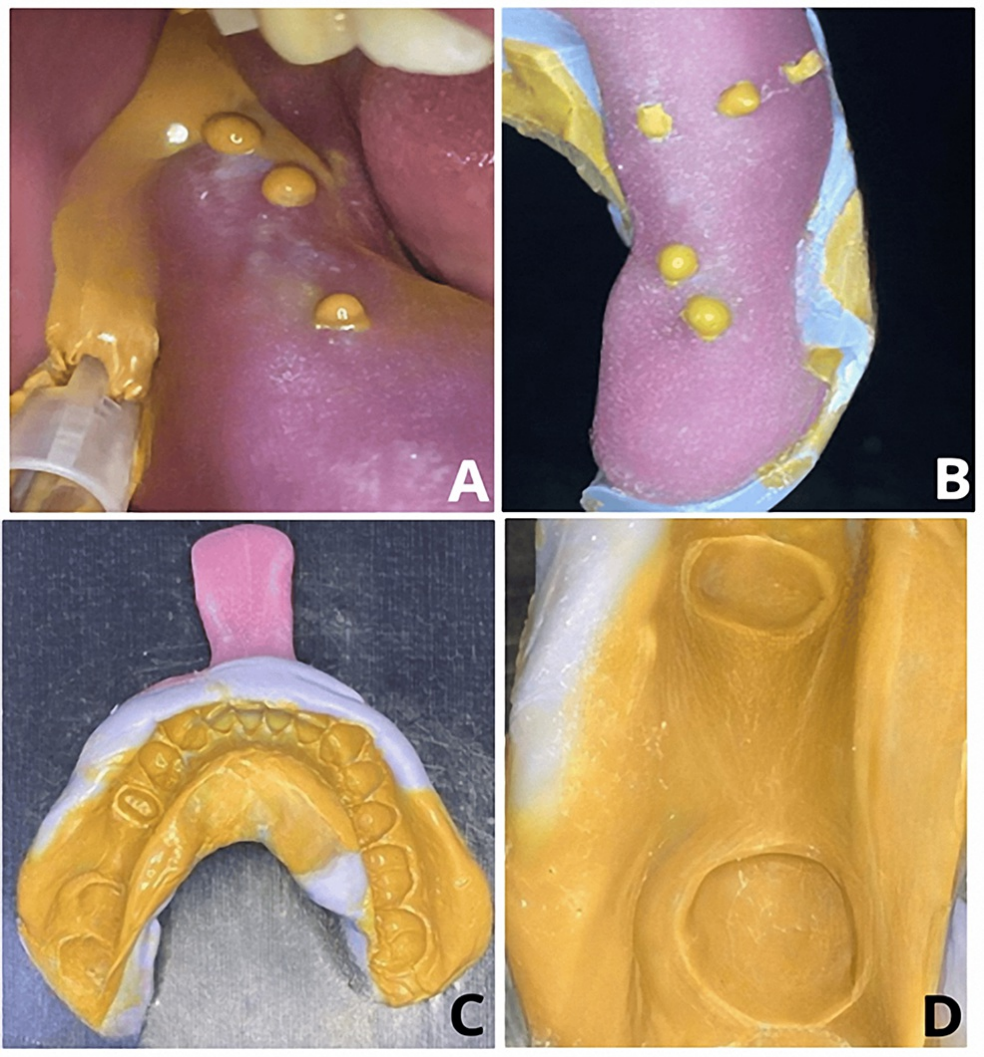
(A) Application of light-body consistency impression final wash material. (B) Notice the escape of the impression material through the holes. (C, D) The final impression with no voids or air bubbles.

13. Create ventilation holes in the putty impression and through the tray using a dental handpiece equipped with a round-end cylinder diamond Bur (Meisinger®) (Figure 3).

14. Fill the tray with a light-body PVS as the final wash.

15. Reposition the tray onto the prepared teeth.

16. Allow excess material to exit through the vent holes (Figure 4).

17. Maintain the tray’s position until the light-body PVS fully polymerizes.

18. Examine the final impression to confirm it is devoid of any air pockets or imperfections (Figure 4).

## Discussion

Several issues have been identified with the two-step impression techniques. First, the effective control of the thickness of the light-body material is lacking. Consequently, in certain marginal impression areas, the putty may displace the light body, leading to the recording of the margin by the putty instead. Second, the composition of the putty consists of materials that possess high elasticity and are prone to alterations when exposed to hydraulic pressure. The consequences of these changes may not manifest until the casting process using the mold has been finalized [8].

According to Hung et al. [3] and Boulton et al. [9], the two-step technique yielded negative discrepancies for both materials, vinyl polysiloxane and polyether, suggesting that smaller dies were produced because of this procedure. The impression material had a significant impact on the two-step technique. Notably, the elevated hardness of vinyl polysiloxane resulted in a 30% reduction in discrepancy in the two-step technique when compared to polyether. However, the nature of the material did not impact the single-step impressions, indicating that the precision of impressions may now be primarily influenced by the technique employed rather than the material itself. This study highlights the significance of the impression technique in the accuracy of dies.

Although other techniques can be executed as single-step impressions, the two-step technique offers several advantages and is well-regarded among clinicians. Most clinicians are well-trained in this method which can be applied even with a stock tray without the need for a special tray. The modified two-step technique presented in this study addresses the issues identified in previous studies. The main advantage of using this modified two-step impression technique is its ease of implementation and simplicity of laboratory procedures. This method helps create an accurate model of the prepared teeth by reducing the pressure from the light-bodied material during the impression-taking process, thus minimizing the potential distortion of the putty mold. Additionally, it is applicable to both single and multiple tooth preparations, especially when the margins are at or below the gum line. However, if the impression tray is not precisely repositioned over the teeth during the wash stage, it may result in an imprecise impression. The methodology described in this study provides a comprehensive explanation of a two-step impression process that involves creating vents in both the tray and the impression material. This approach allows for efficient and accurate capture of the mold of the prepared teeth while reducing overall procedure time.

Limitations

This report outlines a comprehensive methodology for a modified two-step impression technique to obtain accurate impressions of crown margins. Nevertheless, several limitations may impact the effectiveness and precision of the findings. A possible limitation is the intricate nature of the procedure, which necessitates careful execution of multiple steps and may result in errors if not followed diligently. To address this issue, it is recommended that practitioners undergo thorough training and practice to ensure their competence in each step. The investigator underwent comprehensive training and calibration for this study. More specifically, the investigator underwent multiple sessions of training to practice creating holes in the tray at the preparation site using trays that had been previously fabricated and loaded with putty silicone impression material. This repetitive training was conducted to ensure a high level of consistency and accuracy throughout the procedure.

Another limitation is the potential for contamination of materials, such as the putty, by saliva, which could compromise the adhesion and accuracy of the impression. This can be mitigated by employing strict isolation techniques and ensuring a dry working field. Additionally, the use of a custom tray and the need for precise trimming and vent hole placement may increase chair time and material costs. To tackle this issue, professionals can adopt the practice of prepping and tailoring trays in advance, as well as utilizing top-notch materials. This approach will help reduce the probability of errors and the subsequent need for remakes. Additionally, the technique itself may present challenges in consistently achieving the appropriate pressure and alignment during reseating, which could potentially distort the final impression. Therefore, practitioners need to be well-versed in proper tray positioning and the consistent application of even pressure, as this will play a crucial role in minimizing distortion. By adopting these strategies, the limitations associated with the modified two-step impression technique can be effectively managed, thereby leading to more reliable and accurate dental impressions.

## Conclusions

This article introduces a methodology to attain an accurate representation of crown margin preparation for prepared teeth via a two-step impression technique. Through the utilization of a custom tray fabricated from heat-cured acrylic resin and the execution of an initial impression with putty material, subsequently accompanied by the strategic incorporation of vent holes and the application of light-body consistency impression material, this technique effectively streamlines the process. Consequently, this approach minimizes the required time and effort while simultaneously reducing the likelihood of errors commonly observed in conventional two-step impression techniques.
